# The Taiwanese Map of Axial Spondyloarthritis: Living with the Condition

**DOI:** 10.3390/medicina59111962

**Published:** 2023-11-07

**Authors:** Yi-Ning Yen, Marco Garrido-Cumbrera, Yi-Syuan Sun, Chen-Hung Chen, Chien-Chih Lai, Hung-Cheng Tsai, Wei-Sheng Chen, Hsien-Tzung Liao, Yen-Po Tsao, The Ankylosing Spondylitis Caring Society of R.O.C. (ASCARES), Chang-Youh Tsai, Chung-Tei Chou

**Affiliations:** 1 Division of Allergy, Immunology and Rheumatology, Taipei Tzu Chi Hospital, Buddhist Tzu Chi Medical Foundation, New Taipei City 231, Taiwan; larynce@hotmail.com (Y.-N.Y.); kasper4730@hotmail.com (C.-H.C.); 2 Health & Territory Research (HTR), Universidad de Sevilla, 41004 Sevilla, Spain; mcumbrera@us.es; 3 Axial Spondyloarthritis International Federation (ASIF), London WC1N 3AX, UK; 4 Division of Allergy, Immunology & Rheumatology, Taipei Veterans General Hospital, National Yang-Ming Chiao-Tung University, Taipei 112, Taiwan; yssun2@vghtpe.gov.tw (Y.-S.S.); cclai3@vghtpe.gov.tw (C.-C.L.); hctsai7@vghtpe.gov.tw (H.-C.T.); wschen2@vghtpe.gov.tw (W.-S.C.); htliao@vghtpe.gov.tw (H.-T.L.); ctchou@vghtpe.gov.tw (C.-T.C.); 5 Division of Holistic and Multidisciplinary Medicine, Taipei Veterans General Hospital, National Yang-Ming Chiao-Tung University, Taipei 112, Taiwan; dolphinandy@gmail.com; 6 The Ankylosing Spondylitis Caring Society of R.O.C., Taipei 110, Taiwan; 7 Division of Immunology and Rheumatology, Fu Jen Catholic University Hospital, College of Medicine, Fu Jen Catholic University, New Taipei City 243, Taiwan

**Keywords:** axial spondyloarthritis, ankylosing spondylitis, patient-reported outcomes, disease burden, rheumatologist, Taiwan

## Abstract

*Background and Objective*: The International Map of Axial Spondyloarthritis (IMAS) explores the physical, psychological, and social experiences of patients with axial spondyloarthritis (axSpA). This initiative is now being expanded to Taiwan as the Taiwanese Map of Axial Spondyloarthritis (TMAS). We aim to provide rheumatologists with insights into the perspectives of Taiwanese patients, enabling physicians to better understand the unmet needs of these patients and optimize their management. *Materials and Methods*: The TMAS is a cross-sectional study gathering data through an online survey of axSpA patients, promoted by the Ankylosing Spondylitis Caring Society of R.O.C. (ASCARES), conducted from July 2017 to March 2018 by Ipsos, and analyzed by the Health & Territory Research (HTR) group of the University of Seville. The questionnaire includes 99 questions that cover domains such as patient profile, diagnosis, habits/lifestyle, employment status, physical/psychological health status, social support, use of healthcare services, and treatments. *Results*: A total of 112 axSpA patients were included in this survey. The mean age was 38.6 years and 75.0% were male. The average diagnostic delay was 3 years, and 19.6% reported extra-articular manifestations. Out of the 49 respondents who reported HLA-B27 information, 35 were HLA-B27-positive. The disease burden was high, with a mean BASDAI score of 4.9 and 75.9% having a mild to moderate degree of spinal stiffness. Furthermore, they were socially and psychologically burdened, with 88.4% experiencing work-related issues and 25.9% suffering from anxiety. *Conclusions*: The TMAS sheds light on the overall perspective of axSpA patients in Taiwan. The TMAS shows shorter diagnostic delay compared to patients from the EMAS. However, high disease activity and significant psychological distress still trouble the patients, causing functional impairments and even leading to career failures. Understanding the perspective of axSpA patients can help rheumatologists adjust treatment strategies to their unmet needs and improve their disease outcomes.

## 1. Introduction

Axial spondyloarthritis (axSpA) is a chronic inflammatory rheumatic disease affecting more than 20,000 patients in Taiwan [1,2]. It includes ankylosing spondylitis (AS) and non-radiographic axSpA (nr-axSpA).

The European Map of Axial Spondyloarthritis (EMAS) explored the physical, psychological, and social experiences of axSpA patients using a cross-sectional survey including 2846 patients from 13 European countries [3]. To expand this research internationally, the International Map of Axial Spondyloarthritis (IMAS) survey, which included Taiwan, was conducted, completing the Taiwan Map of Axial Spondyloarthritis (TMAS). The TMAS not only gathers the perspectives of Taiwanese axSpA patients with regards to the management of their disease but also their experience on how the disease influences their lives. To the best of our knowledge, this is the first study in Taiwan that has attempted to collect patient-reported outcomes of axSpA; it has offered physicians insights into Taiwanese patients’ perspective on their disease.

## 2. Materials and Methods

Similar to the EMAS, the TMAS was a cross-sectional study collecting data through a survey of axSpA patients from all over Taiwan. The TMAS gathered data through an online survey of axSpA patients, promoted by the Ankylosing Spondylitis Caring Society of R.O.C. (ASCARES), conducted from July 2017 to March 2018 by Ipsos, and analyzed by the Health & Territory Research (HTR) group of the University of Sevilla with support from Novartis Ltd., Taipei, Taiwan. The ASCARES is a patient support group that consists of patients aged 20 years or older with either AS or axSpA, or legal guardians of patients under 20 years old. It provides its members with health education information and mental support and organizes outdoor activities. There were about 470 members in total during the time this survey was conducted.

The questionnaire contained 99 items within 13 sections, including a profile of the survey participants, diagnosis, physical health, psychological health, social support, healthcare, pharmacological treatment, habits and lifestyle, employment status, and fears and hopes. The survey also included the following validated scales:

Bath Ankylosing Spondylitis Disease Activity Index (BASDAI) [4]: a widely used scale for the assessment of disease activity in axSpA research. It is comprised of six items assessing fatigue, pain, overall discomfort, and morning stiffness severity and duration. The mean score of the items results in a scale ranging from 0 to 10. A BASDAI value of 4 and above indicates high disease activity.

Twelve Item General Health Questionnaire (GHQ-12) [5]: a brief questionnaire for the assessment of psychological distress in both clinical and general populations. It is comprised of twelve 4-point Likert-scale items. For the purpose of this study, items were dichotomized, with values 0 and 1 converted to 0 and 2 and 3 converted to 1, resulting in a scale ranging from 0 to 12. The threshold was situated at 3, with values above or equal indicating psychological distress.

The survey was posted online from July 2017 to March 2018. Participants included many members of the ASCARES, which aided in distributing information about the research and helped in recruiting participants. The patients involved in the current study were all aged 18 years or more and the diagnosis of axSpA was self-reported. Patients were not eligible to participate in the present study if they were under 18 years old or if they were not a resident in Taiwan. All participants signed an opt-in consent form prior to survey participation. All the data collected were anonymized.

Descriptive statistics was mainly used throughout this study. We summarized the information collected to determine the central tendency and distribution of the gathered data.

## 3. Results

**Figure 1:**
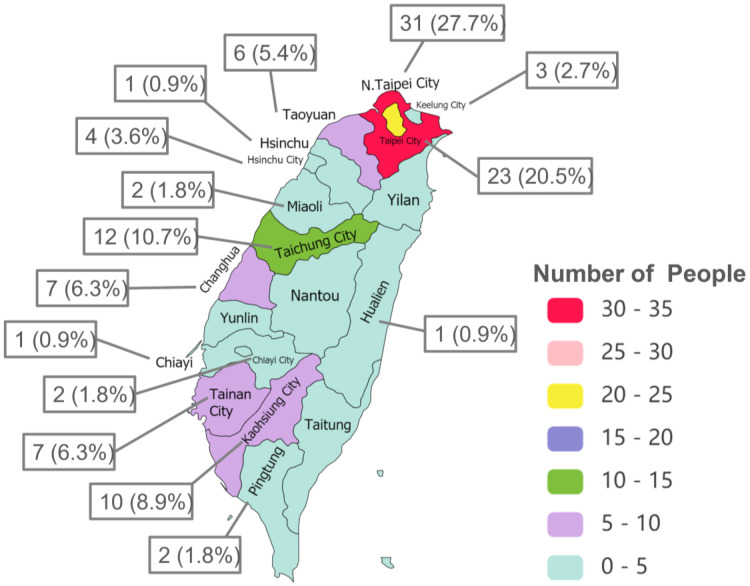
Residency regions of the participants in Taiwan (*n* = 112), showing that the participants reside in different parts of Taiwan but most are from northern Taiwan.

The TMAS survey was completed by 112 patients with axSpA from 15 regions of Taiwan. Most of the participants were from New Taipei City (formerly Taipei County), Taipei Metropolis, Taichung Metropolis, and Kaohsiung Metropolis (Figure 1). The participants were between 18 and 67 years of age, and the mean age was 38.6 years. Three out of four patients were male. Also, most of the patients were married (59.8%) and with a university degree (83.9%). The body mass index (BMI) of more than half of these participants (52.7%) was between 18.5 and 24.9, and 37.5% of them had a BMI of over 25. Non-smokers account for 69.6% of the 112 participants, and almost 85% of them had never or occasionally consumed alcohol. Around 40% of the patients belonged to a patient support group (Table 1).

Table 2 shows that the mean age of symptom onset was 28.1 years, the mean age at diagnosis was 31 years, and the mean diagnostic delay was 3 years. Also, 22 participants (19.6%) reported an extra-articular manifestation such as uveitis or inflammatory bowel disease. Of the 49 patients who reported their human leukocyte antigen (HLA)-B27 status, 35 said that they were HLA-B27-positive. The mean BASDAI score was 4.9, and most patients (75.9%) had a mild to moderate degree of spinal stiffness.

The patients’ employment status, work-related issues, psychological health, and disease-related perceptions are summarized in Table 3. With respect to the labor force, 87 patients (96.7%) were employed, compared to only 3 unemployed (3.3%). Ninety-nine patients (88.4%) experienced work-related issues because of axSpA. This included missing work for doctor’s appointment, taking sick leaves, difficulties in job searching, and job choice considerations because of axSpA. About a quarter of these participants (25.9%) suffered from anxiety. Other psychological problems such as sleep disorders (23.2%) and depression (20.5%) were also reported. The mean score of the GHQ-12 used in assessing the psychological health of the participants was 3.3, with about 45% of the 112 patients having a score of more than 3.

Participants stated that their most common fears were suffering pain (70.3%), spinal stiffness (67.6%), and disease progression (57.7%). They reported hopes to eliminate pain and spinal stiffness and reduce disease flares. The three most common treatment goals they had were to eliminate pain (70.5%) and reduce stiffness (67.0%) and flares (58.9%). Nevertheless, only 37% of the participants had talked with their treating physician about treatment goals.

## 4. Discussion

The TMAS was the first patient survey in Taiwan to emphasize the personal experience and opinions of axSpA patients, highlighting high disease activity among Taiwanese patients with consequences for their physical as well as mental health. The participants reported that their lives were limited by the symptoms of axSpA and the inconvenience that came with it. This led to lifestyle adjustments, employment issues, and career stagnation. The healthcare of axSpA in Taiwan included reimbursement for non-steroidal anti-inflammatory drugs (NSAIDs), but to apply for reimbursement of biologics, patients had to test positive for HLA-B27 and their C-reactive protein (CRP) and erythrocyte sedimentation rate (ESR) had to be more than 1 mg/dL and 28 mm/h, respectively, which largely limited biologics’ use in Taiwanese axSpA patients.

The mean body mass index (BMI) of TMAS participants was 24 kg/m^2^, while the normal BMI for the general Taiwanese population is between 18.5 and 24 kg/m^2^. About 38% participants in our study had a BMI ≥ 25, a percentage compatible with the proportion of the overweight (BMI ≥ 24) population in Taiwan [6]. There were 78 non-smokers (69.6%) in our cohort, which is in line with another study conducted on axSpA patients in Taiwan [7]. Smoking is generally inversely correlated with being overweight [8], and the relationship of smoking axSpA patients with higher BMI in the present study deserves further investigation.

Most of the participants (84%) in the TMAS survey had never or only occasionally consumed alcohol. It seemed that disease activity (BASDAI score) and the frequency of alcohol consumption had no definite association in our study (*p* = 0.417), similar to the finding in a recent study of Taiwanese axSpA patients [7].

The mean age at onset of symptoms was 26.6 ± 11.0 years and the mean age at diagnosis was 34.1 ± 11.1 years in the EMAS study [9]. The resulting mean diagnostic delay in this regard was 7.4 ± 8.4 years in the EMAS study. Overall, the diagnostic delay of Taiwanese axSpA patients (3.0 ± 5.6 years) in this study was far shorter than that in the EMAS. A possible reason for the shorter diagnostic delay noted in the present study may be the readily available and easily accessible medical services in small territorial Taiwan, especially in metropoles like New Taipei City, Taipei, Taichung, and Kaohsiung, where most of the healthcare resources are concentrated [10]. Also, awareness of rheumatic diseases in the general population is high because of the health educational programs frequently carried out by hospital-aided patient organizations and provided through mass communication media.

Additionally, diagnostic delay was longer in males, which was in contrast with the EMAS survey, which found the diagnostic delay to be 8.2 ± 8.9 years for females and 6.1 ± 7.4 years for males [11]. Another systematic review and meta-analysis by Jovaní et al. (2016) also found gender differences in diagnostic delay, i.e., a mean of 8.8 years (7.4–10.1) for women and 6.5 (5.6–7.4) for men (*p* = 0.01). The mean diagnostic delay was 6.6 years [12]. A possible reason for the shorter diagnostic delay in females that was found in TMAS survey may be the smaller percentage of women included in this cohort (only 25%) compared to the figure reported in the systematic review and meta-analysis by Jovaní et al. (2016), which included 23,883 patients, of whom 32.3% were women [12], and the EMAS survey [3], in which females accounted for 61.4% of all patients.

The TMAS survey showed that in the 112 participants, up to 51 (45.9%) had their axSpA diagnosed by orthopedic specialists and only 40 (36%) were diagnosed by rheumatologists. A study by Danve et al. (2019) on axSpA patients in the USA reported similar results, with only 37% of patients with AS diagnosed by rheumatologists [13]. Also, the TMAS survey explored the healthcare professionals visited by these patients before axSpA was diagnosed and found that up to 68.6% of the participants had seen an orthopedic specialist, 48.6% had seen a general practitioner, but only 28.6% had seen a rheumatologist.

For these 112 axSpA patients, less than 50% had their disease managed by a rheumatologist. About 40% were being handled by an orthopedic specialist or a physiotherapist rather than a rheumatologist. What’s more, the patients frequently reported that they had visited more than one specialist before their diagnosis of axSpA was confirmed, indicating that their path to diagnosis was not optimal and they were not referred to a rheumatologist directly.

This is a major concern and an unmet need for patients with axSpA as rheumatologists should be the main medical specialist in charge of these patients. There are around 360 rheumatologists in Taiwan, but more than 1500 orthopedic specialists, and over 9000 general practitioners [14]. This reflects the fact that these primary healthcare providers play a rather important role in identifying suspected axSpA cases and referring them to a rheumatologist. This situation might have contributed to the diagnostic delay these patients experienced since most of the patients were not managed by rheumatologists before they were diagnosed with axSpA, further emphasizing the need to advocate to primary healthcare providers the need for holistic management of axSpA patients and the timing of referral of such patients to a rheumatologist.

The results of the TMAS showed high disease activity, with more than 70% of the patients having a BASDAI score equal to or greater than 4. The high disease burden observed in these individuals could have been due to several reasons, like delayed diagnosis or sub-optimal pharmacological treatment for disease activity control. Also, a high proportion of patients made compromises in their life, such as customized shoes (59.8%) and home adjustments (51.8%). This most likely resulted from their high disease burden, forcing them to make certain modifications in their lifestyles.

The TMAS survey disclosed that less than 20% of the participants received biologics. On the other hand, a study utilizing a research database in the US found that 39.3% of the patients with AS were given biologics [15]. Given the Taiwanese participants’ relatively high disease burden, the percentage of biologics users seems low. The reason for this low usage might have been the National Health Insurance (NIH) of Taiwan’s restrictions on the reimbursement of biologics. Patients must test positive for HLA-B27 and their C-reactive protein (CRP) and erythrocyte sedimentation rate (ESR) have to be more than 1 mg/dL and 28 mm/h, respectively. This limits the access to biologics in Taiwanese patients with axSpA.

The impact axSpA poses on life is more than just functional limitations or making adaptations. The participants’ work life seemed to be affected, as well. Patients were somehow forced to make workplace adaptations (58.9%) and even change their jobs (42.9%). In this survey, the mean BASDAI score was significantly higher in participants whose professional life had suffered. Patients with a higher BASDAI score generally had more physical comorbidities and a higher level of functional limitation. In a Dutch study, the BASDAI score was a predictor of adverse work outcomes over a period of 12 years [16]. Another study describing the influence of AS on sick leave, presenteeism, and limitations in physical function when carrying out unpaid tasks associated those with unsatisfactory work outcomes [17]. Poor performance at work can lead to an unsuccessful career, thus placing a financial burden on the patients and their families, or even on society [18]. A previous study conducted in the Netherlands in 2001 showed that withdrawal from the labor force because of AS-related work disability was 5% during the first year after diagnosis, 13% after 5 years, and 31% after 20 years [19]. This could also mean early retirement for axSpA patients.

Psychological health is another issue that burdens the patients with axSpA. Previous studies found that disturbed sleep was a rather prevalent and significant aspect of the disease. In addition to depression and anxiety, higher disease activity was associated with poorer sleep quality [20].

In the present study, 44.6% of the participants scored higher than or equal to 3 on the GHQ-12, which indicated a risk of psychological distress. These results are in line with a recent Taiwanese study that found the risk of having depression, anxiety, or sleep disorder was higher in AS patients [21]. Another meta-analysis of 16 axSpA studies revealed that the prevalence of depression among these patients was around 11–64% [22]. Our results, as well as the aforementioned studies, suggest that axSpA patients face more psychological distress than the general population. The disease not only affects the patients in their physical well-being but also disturbs their mental health.

There are some limitations in the present study. First, the sample size was small. Second, like the EMAS, this study relied on patient-reported data, with no confirmation of participant diagnosis and no physician-reported assessments. Third, the questionnaire was translated by non-physicians and not verified by any clinicians, and due to translational inaccuracies in the question regarding the patients’ condition, nr-axSpA patients may not have been included in this survey. Fourth, because the survey was distributed online, patients who had limited access to the internet (living in suburban areas, incapability of using smartphones or computers, etc.) were unable to join the study. Moreover, clinicians were not involved in the collection of these data, and a lot of participants were recruited by the patient support group, the ASCARES in Taiwan, leading to selection bias.

Nonetheless, the TMAS is the first survey conducted in Taiwan aiming to understand the perspective of axSpA patients using a holistic approach. To do so, the research heavily relies on patient research partners and on collecting data through a questionnaire made for patients by patients, with the purpose of reflecting axSpA patients’ real needs. This methodology has been and is being widely implemented around the world as more and more countries in America, Asia, and Africa are joining the IMAS project, and its results have already supported patient advocacy movements in Europe.

## 5. Conclusions

The present study has provided a description of the baseline characteristics of axSpA patients in Taiwan. Taiwanese patients showed shorter diagnostic delay compared to that of the European sample, but higher disease activity and significant physical and psychological burdens, causing functional impairment and even leading to career failures. Standing in axSpA patients’ shoes, knowing their weaknesses, fears, and hopes, and hearing their thoughts on treatment goals can help rheumatologists understand their point of view and adjust treatment strategies accordingly.

## Figures and Tables

**Table 1:** Socio-demographic, lifestyle, and anthropometric variables.

Variable, *n*	Mean ± SD/*n* (%)
Age (years), *n* = 112	38.6 ± 10.3
18–34	39 (34.8%)
35–51	60 (53.6%)
52–68	13 (11.6%)
Gender (female), *n* = 112	28 (25%)
Marital status, *n* = 112	
Single	40 (35.7%)
Married	67 (59.8%)
Divorced/separated	4 (3.6%)
Widowed	1 (0.9%)
Educational level, *n* = 112	
Primary school	1 (0.9%)
High school	17 (15.2%)
College and university	94 (83.9%)
Monthly income (New Taiwan Dollars) ^†^, *n* = 107	22,971 ± 16,030
Body mass index (BMI), *n* = 112	
Underweight (BMI < 18.5)	11 (9.8%)
Normal weight (BMI 18.5–24.9)	59 (52.7%)
Overweight (BMI 25.0–29.9)	28 (25%)
Obesity (BMI ≥ 30.0)	14 (12.5%)
Smoking, *n* = 112	
Non-smoker	78 (69.6%)
Smoker	34 (30.4%)
Alcohol consumption, *n* = 112	
Never	34 (30.4%)
Occasionally	60 (53.6%)
1 time per week	8 (7.1%)
2–3 days per week	8 (7.1%)
4–5 days per week	1 (0.9%)
Every day	1 (0.9%)
Member of a patient support group, *n* = 112	
Yes	44 (39.3%)

^†^ New Taiwan Dollar 27.9 = USD 1.00.

**Table 2:** Disease-specific characteristics.

Variable, *n*	Mean ± SD/*n* (%)
Age at onset of the 1st symptoms, years, *n* = 112	28.1 ± 10.3
Age at diagnosis, years, *n* = 112	31 ± 10.4
Male (*n* = 84)	30.5 ± 10.6
Female (*n* = 28)	32.5 ± 9.9
Diagnostic delay, years, *n* = 112	3 ± 5.6
Male (*n* = 84)	3.6 ± 6.2
Female (*n* = 28)	1.3 ± 2.5
Disease duration, years, *n* = 112	
Male (*n* = 84)	12.3 ± 10.6
Female (*n* = 28)	4.8 ± 4.2
Extra-articular manifestations, *n* = 112	
Uveitis	9 (8.0%)
Crohn’s disease	6 (5.4%)
Ulcerative colitis	7 (6.3%)
HLA-B27 (positive), *n* = 49	35 (71.4%)
^†^ BASDAI (0–10), *n* = 112	4.9 ± 1.9
BASDAI < 4	32 (28.6%)
BASDAI ≥ 4	80 (71.4%)
Spinal stiffness, *n* = 112	
No stiffness	9 (8.0%)
Mild	52 (46.4%)
Moderate	33 (29.5%)
Severe	18 (16.1%)

^†^ BASDAI: Bath Ankylosing Spondylitis Disease Activity Index.

**Table 3:** Career-related aspects, psychological health, and perception of disease.

Variable, *n*	Mean ± SD/*n* (%)
Employment status of labor force, *n* = 90	
Employed	87 (96.7%)
Unemployed	3 (3.3%)
Left/lost job because of axSpA, *n* = 3	2 (66.7%)
Employment status of the economically inactive, *n* = 22	
Retired	6 (27.3%)
Temporary sick leave	6 (27.3%)
Permanent sick leave	3 (13.6%)
Homemaker	3 (13.6%)
Early retirement	2 (9.1%)
Student	1 (4.5%)
Other	1 (4.5%)
Work-related issues due to axSpA, *n* = 112	99 (88.4%)
Missed work for doctor’s appointment, *n* = 96	47 (49.0%)
Took sick leave because of axSpA, *n* = 96	34 (35.4%)
Difficulties finding a job due to axSpA, *n* = 103	51 (49.5%)
AxSpA influenced job choice, *n* = 106	59 (55.7%)
Psychological comorbidities, *n* = 112	
Anxiety	29 (25.9%)
Sleep disorder	26 (23.2%)
Depression	23 (20.5%)
GHQ-12 ^†^ score (0–12), *n* = 112	3.3 ± 3.4
GHQ-12 ≥ 3	50 (44.6%)
GHQ-12 < 3	62 (55.4%)
Most common fears, *n* = 111	
Suffering pain	78 (70.3%)
Suffering stiffness	75 (67.6%)
Disease progression	64 (57.7%)
Suffering fatigue	63 (56.8%)
Most common hopes, *n* = 112	
Eliminate pain	78 (69.6%)
Eliminate stiffness	74 (66.1%)
Reduce flares	68 (60.7%)
Reduce fatigue	67 (59.8%)
Most common treatment goals, *n* = 112	
To eliminate/reduce pain	79 (70.5%)
To eliminate/reduce stiffness	75 (67.0%)
To reduce flares	66 (58.9%)
Halt disease progression	61 (54.5%)
Talked with treating physician about treatment goals, *n* = 108	40 (37.0%)

^†^ GHQ-12: 12 Item General Health Questionnaire.

## Data Availability

Data are contained within the article.
